# Dynamics of Elliptical Magnetic Skyrmion in Defective Racetrack

**DOI:** 10.3390/nano14030312

**Published:** 2024-02-04

**Authors:** Huangkun Zhu, Gang Xiang, Youhua Feng, Xi Zhang

**Affiliations:** College of Physics, Sichuan University, Chengdu 610065, China

**Keywords:** elliptical skyrmion, defect, racetrack, dynamics, micromagnetic simulation

## Abstract

Recently, it has been reported that the skyrmion Hall effect can be suppressed in an elliptical skyrmion-based device. Given that defects are unavoidable in materials, it is necessary and important to investigate the dynamics of an elliptical skyrmion in a defective racetrack device. In this work, the current-driven dynamics of an elliptical skyrmion in a defective racetrack device are systematically studied using micromagnetic simulations. The system energy analysis reveals that the magnetic parameters of the circular defect play critical roles in determining the type (repulsive or attractive) and the magnitude of the force on the elliptical skyrmion. The simulated trajectories show that the primary motion modes of the elliptical skyrmion in the defective racetrack can be divided into four types, which are dependent on the values of the Dzyaloshinskii–Moriya interaction (DMI) constant *D_d_*, the perpendicular magnetic anisotropy constant *K_d_*, the magnitude of the driving current density *J*, and the size *d* of the defect. Further investigation of the motion-mode phases of the skyrmion reveals the synthetic effects of *D_d_*, *K_d_*, *J,* and *d*. Finally, the minimum depinning current density *J*, which linearly depends on the parameters of *D_d_* and *K_d_*, is obtained for a skyrmion completely pinned in the defect. Our findings give insights into the dynamics of an elliptical skyrmion in the presence of a defect with different magnetic parameters in a racetrack device and may be useful for performance enhancement of skyrmion-based racetrack memory devices.

## 1. Introduction

Magnetic skyrmion, a topological magnetic structure characterized by a nanometric size and remarkable stability, has gained significant attention since its first experimental observation in 2009 [1,2,3]. Magnetic skyrmion can be used for various spintronic devices, including spin logic gates [4,5], transistor-like devices [6,7,8], spin diodes [9,10,11], spin-torque nano-oscillators [12,13,14], and racetrack memory devices [15,16]. In particular, racetrack memory devices based on a magnetic skyrmion offer the advantages of high storage density and low energy consumption. However, the presence of the skyrmion Hall effect, which causes the transverse motion of a magnetic skyrmion, hinders the smooth movement of the skyrmion along the track and leads to the annihilation of the skyrmion at the track edge [17], resulting in instability and even failure of related devices. It has been reported that the hetero DMI interfaces [18,19,20] and vortex–skyrmion interaction in superconductor–chiral magnet bilayers [21] could offer solutions for the unwanted skyrmion Hall effect. Additionally, recent studies have shown that shape anisotropy can serve as an alternative means of controlling the motion direction of a magnetic skyrmion [22,23,24]. Notably, owing to its unconventional shape anisotropy [25,26,27], it has been reported that an elliptical skyrmion can also suppress the skyrmion Hall effect. Simulations have shown that the dynamics of an elliptical skyrmion differs significantly from that of a circular skyrmion [28], as an elliptical skyrmion can move along a track without transverse motion [29]. Interestingly, although the influence of defects on the motion of a circular skyrmion has been extensively studied [30,31], the impact of defects on the current-driven motion of an elliptical skyrmion has not been reported. Given that defects are unavoidable in materials, it is necessary and important to investigate the dynamics of an elliptical skyrmion in a defective racetrack device.

In this work, the interaction between an elliptical skyrmion and a defect in a racetrack is investigated using micromagnetic simulations. Our results show that, dependent on the Dzyaloshinskii–Moriya interaction (DMI) and the perpendicular magnetic anisotropy (PMA) constants of the defect in the racetrack, the elliptical skyrmion feels different types (repulsive or attractive) and magnitude of forces and, hence, exhibits different motions. The influence of defect size and driving current density on the dynamics of the elliptical skyrmion is also investigated. In addition, since the pinning and depinning of a normal-shaped skyrmion has been investigated [32] while that of an elliptical skyrmion is still unclear, the pinning and depinning of the elliptical skyrmion is also studied. This study provides a theoretical guidance for the potential application of elliptical skyrmions in racetracks.

## 2. Method

**Figure 1:**
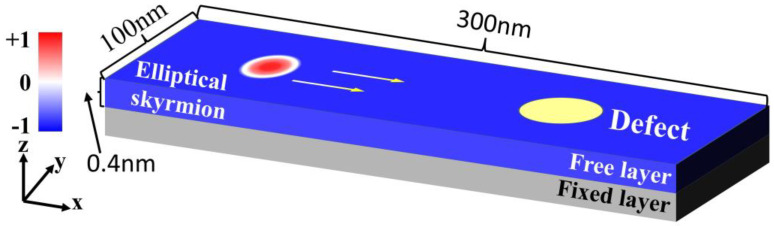
The schematic of the proposed defective racetrack device.

The proposed structure comprises two main components: a free layer and a fixed layer, as shown in Figure 1. The free layer is a Co/Pt bilayer racetrack measuring 300 nm in length, 100 nm in width, and 0.4 nm in thickness, with a circular defect located on the right side. To achieve the formation of an elliptical skyrmion, anisotropic DMI is introduced by introducing C_2v_ symmetry [33]. Since the defect may be caused by different impurities or inhomogeneity [34,35,36], different parameters of the defect are simulated, including the DMI and PMA constants and the diameter of the defect. The fixed layer features a polarized magnetization structure oriented at an angle of 70° relative to the y-axis to make the elliptical skyrmion move along a straight line. The elliptical skyrmion within the free layer can be propelled by injecting electric current along the z-axis, a phenomenon that is driven by the spin-transfer torque (STT) effect [37].

Our micromagnetic simulations are conducted using the Object-Oriented Micromagnetic Framework (OOMMF) software version 2.0 beta 0, which includes extension modules for the anisotropic DMI and spin-transfer torques. The OOMMF is widely used in micromagnetic simulations, as is Mumax3 [38]. The current-driven skyrmion dynamics is investigated by numerically solving the Landau–Lifshitz–Gilbert (LLG) equation with spin-transfer torques as follows:

(1) $$\begin{array}{c} \frac{dm}{dt}=-\gamma_{0}m\times H_{eff}+\alpha \left(m\times \frac{dm}{dt}\right)+u\left(m\times m_{p}\times m\right) \end{array}$$

where $m=\frac{M}{M_{s}}$, *M_s_* is the saturation magnetization, *γ*_0_ is the electron gyromagnetic ratio, *α* is the damping coefficient, $u=\frac{\gamma_{0}\hbar JP}{2\mu_{0}eM_{s}d_{t}}$, *ħ* is the reduced Planck constant, *J* is the current density, *P* is the spin polarization ratio, *e* is the electron charge, *μ*_0_ is the vacuum permeability, *d_t_* is the thickness of the Co layer, and ***H_eff_*** is the effective field including the Heisenberg exchange, the anisotropic Dzyaloshinskii–Moriya interaction (DMI), the PMA, and the demagnetization. The discretization cell size is set as 1 × 1 × 0.4 nm^3^. The material parameters used in this work are adopted from Ref. [39]: the saturation magnetization *M_s_* = 0.58 MA/m, the Heisenberg exchange stiffness constant *A* = 15 pJ/m, the PMA constant *K_u_* = 0.8 MJ/m^3^, the damping coefficient *α* = 0.3, and the DMI constants in the *x*-direction *D_x_* = 3.7 mJ/m^2^ and in the *y*-direction *D_y_* = 2.5 mJ/m^2^. The parameters of the circular-shaped defect with a diameter of 10–40 nm are as follows: in the case of a non-magnetic defect, which could be due to a non-magnetic impurity material or even a vacancy, all the magnetic parameters are zero; in the case of a magnetic defect, which could be due to a magnetic impurity material or a defective racetrack material with various types of dopants, the DMI constant of the defect is represented as *D_d_*, which ranges from 1.0 to 4.5 mJ/m^2^, and the PMA constant of the defect is represented as *K_d_*, which varies from 0.5 to 1.1 MJ/m^3^. The applied current density *J* in the racetrack is 5–20 × 10^10^ A/m^2^. 

## 3. Results 

**Figure 2:**
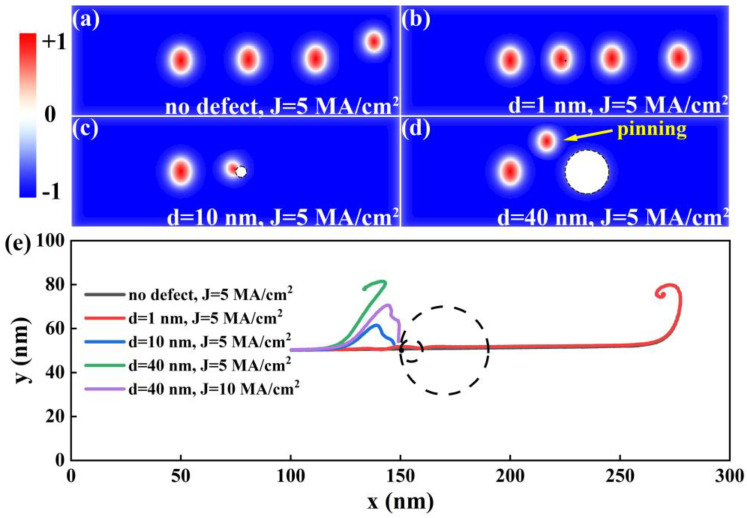
The trajectories of an elliptical skyrmion in the absence of a defect and in the presence of a non-magnetic defect with different diameters *d*. The parameters of the defect and the snapshot time information of the skyrmion from the left to the right are (**a**) no defect, with t = 0, 2 ns, 4 ns, and 6 ns; (**b**) *d* = 1 nm, with t = 0, 1.5 ns, 3 ns, and 5 ns; (**c**) *d* = 10 nm, with t = 0 and 2 ns; (**d**) *d* = 40 nm, with t = 0 and 10 ns; and (**e**) the whole trajectories of the elliptical skyrmion center within 10 ns, in which the black circle represents the position of a vacancy defect.

We first studied the motion of an elliptical skyrmion in the situation when there is a non-magnetic defect in the racetrack. For comparison, the situation without a defect is also simulated, as shown in Figure 2a. It is evident that the elliptical skyrmion moves almost straight in a racetrack without a defect. When a defect is introduced, the motion of the elliptical skyrmion is dependent on the size of the defect. As shown in Figure 2b–d, when the diameter (*d*) of the defect increases from 1 to 40 nm with the current density remaining at 5 MA/cm^2^, the motion of the elliptical skyrmion changes dramatically. In the case of *d* = 1 nm, the elliptical skyrmion, which is around tens of nanometers, can go across the defect directly since the influence of the defect on the elliptical skyrmion is insignificant owing to the defect’s tiny size. In the case when the defect’s diameter increases to 10 nm, the elliptical skyrmion gets annihilated at the position of the defect, indicating that the contact between the elliptical skyrmion and the non-magnetic defect can cause the annihilation of the elliptical skyrmion. In the case of *d* = 40 nm, the elliptical skyrmion is pinned at the top-left exterior of the defect, indicating that the defect shows a repulsive force, and the repulsive force is large enough to prevent the elliptical skyrmion from approaching the defect. The whole trajectories of the elliptical skyrmion center under the influence of different sizes of the nonmagnetic defect are shown in Figure 2e. Additionally, the influence of the magnitude of the electric driving current density *J* on the trajectory of the elliptical skyrmion is also investigated and shown in Figure 2e, where *J* increases from 5 to 10 MA/cm^2^ and the diameter of the non-magnetic defect remains at 40 nm. When *J* increases up to 10 MA/cm^2^, the elliptical skyrmion can approach the defect and gets annihilated in it since the increased driving force can overcome the repulsive force from the defect.

**Figure 3:**
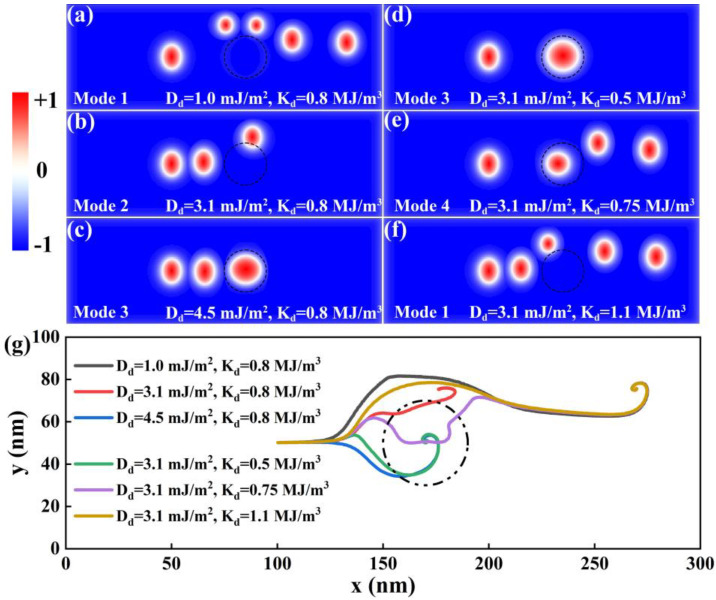
The trajectories of an elliptical skyrmion in the presence of a magnetic defect with different *D_d_* and *K_d_* values. The parameters of the defect and the snapshot time information of the skyrmion from left to right are (**a**) *D_d_* = 1.0 mJ/m^2^, with t = 0, 2 ns, 5 ns, 6 ns, and 8 ns; (**b**) *D_d_* = 3.1 mJ/m^2^, with t = 0, 1 ns, and 10 ns (pinning position); (**c**) *D_d_* = 4.5 mJ/m^2^, with t = 0, 1 ns, and 10 ns (pinning position); (**d**) *K_d_* = 0.5 MJ/m^3^, with t = 0 and 10 ns (pinning position); (**e**) *K_d_* = 0.75 MJ/m^3^, with t = 0, 2 ns, 4 ns, and 6 ns; and (**f**) *K_d_* = 1.1 MJ/m^3^, with t = 0, 1 ns, 2 ns, 4 ns, and 6 ns. The black circle with a diameter of 40 nm represents the defect position. (**g**) The whole trajectories of the elliptical skyrmion center within 10 ns.

Next, we studied the dynamics of the elliptical skyrmion when the defect in the racetrack is magnetic. In this situation, the magnetic parameters of the defect are nonzero, and the motion of the elliptical skyrmion was investigated by gradually varying the DMI constant *D_d_* and the perpendicular magnetic anisotropy constant *K_d_* of the defect. Firstly, we gradually increased *D_d_* of the defect from 1.0 to 4.5 mJ/m^2^, with *K_d_* remaining the same as *K_u_* (0.8 MJ/m^3^) of the racetrack and the electric current remaining at a medium level of 5 MA/cm^2^. This situation can be caused by an impurity atom. The simulated results are shown in Figure 3a–c. One can find that, as *D_d_* increases, the motion mode of the elliptical skyrmion changes from bypassing the defect from the side to being pinned at the top-right interior of the defect, and then to being pinned at the center of the defect, owing to the change in the interaction between the elliptical skyrmion and the defect. When *D_d_* is smaller than *D_y_* (2.5 mJ/m^2^) of the racetrack, the defect shows a repulsive force on the elliptical skyrmion, and the elliptical skyrmion can bypass the defect (Figure 3a). When *D_d_* is bigger than *D_y_* (2.5 mJ/m^2^) but still smaller than *D_x_* (3.7 mJ/m^2^) of the racetrack, the skyrmion can approach and finally get pinned at the top-right interior of the defect (Figure 3b). When *D_d_* is large enough, the elliptical skyrmion enters the defect center and gets pinned at the defect center. In this case, it is unknown at present whether the defect shows an attractive force or a small repulsive force on the skyrmion outside the defect. Notably, when the elliptical skyrmion is pinned at the defect center, the shape of the skyrmion will become circular due to the isotropic DMI of the defect.

Then, we gradually increased *K_d_* of the defect from 0.5 to 1.1 MJ/m^3^, with the electric current remaining at 5 MA/cm^2^ and the DMI of the defect remaining at 3.1 mJ/m^2^, which is close to the average values of *D_x_* and *D_y_* of the racetrack. When *K_d_* is much smaller than *K_u_* (0.8 MJ/m^3^), the elliptical skyrmion enters the defect and gets pinned at the defect center, as shown in Figure 3d. At present, it is unknown, in this case, whether the defect shows an attractive force or a small repulsive force on the skyrmion outside the defect. When *K_d_* increases to be close to but still smaller than *K_u_*, the elliptical skyrmion can go through the defect, as shown in Figure 3e, probably because the deference in *K_d_* between the defect and the racetrack gets smaller. When *K_d_* is much larger than *K_u_*, the elliptical skyrmion can bypass the defect, as shown in Figure 3f, which is very similar to the case of *D_d_* = 1.0 mJ/m^2^ (Figure 3a), indicating that the defect shows a repulsive force on the skyrmion outside the defect.

Additionally, in order to present the whole motion trajectory of the elliptical skyrmion under the influence of different DMIs and perpendicular magnetic anisotropy constants, the center point of the elliptical skyrmion in every step was calculated and is shown in Figure 3g. According to the discussion above, the motion modes of an elliptical skyrmion in a defective racetrack can be divided into four types, which are Mode 1 (Figure 3a,f), Mode 2 (Figure 3b), Mode 3 (Figure 3c,d), and Mode 4 (Figure 3e). In the following section, we will use Modes 1–4 to refer to these four motion modes of the elliptical skyrmion for convenience. 

**Figure 4:**
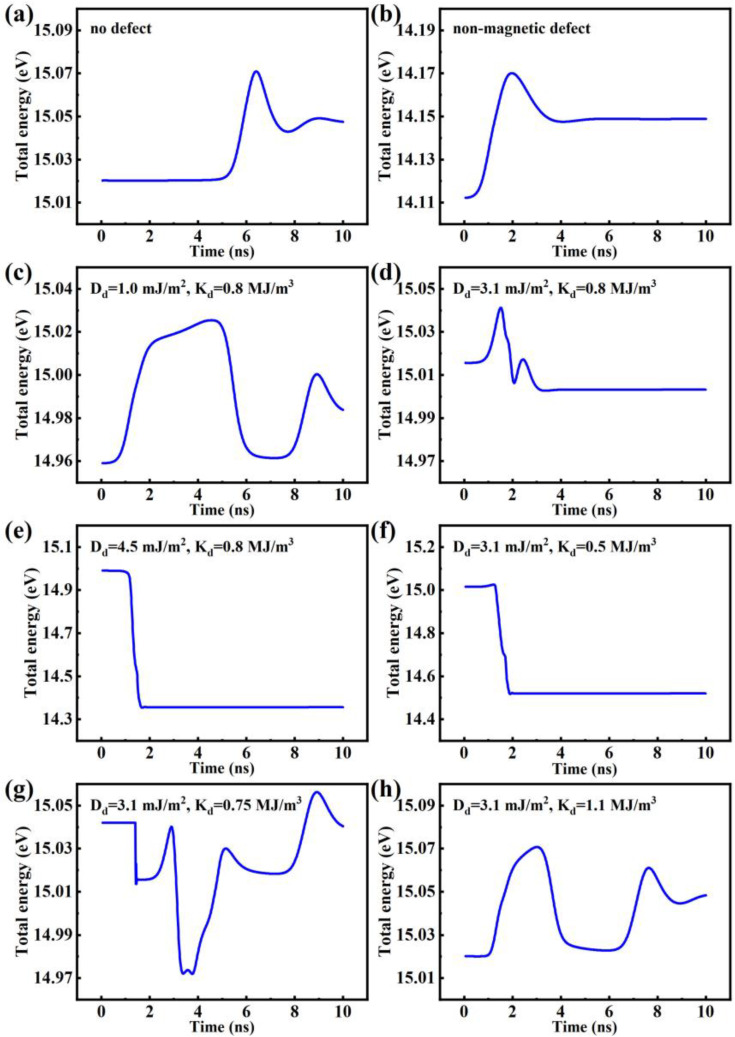
The system energy of every step time (**a**) when there is no defect, (**b**) in the case of a non-magnetic defect, and in the case of a magnetic defect with (**c**) *D_d_* = 1.0 mJ/m^2^, (**d**) *D_d_* = 3.1 mJ/m^2^, (**e**) *D_d_* = 4.5 mJ/m^2^, (**f**) *K_d_* = 0.5 MJ/m^3^, (**g**) *K_d_* = 0.75 MJ/m^3^, or (**h**) *K_d_* = 1.1 MJ/m^3^, in which the diameter of the defect is 40 nm and the driving current density remains at 5 MA/cm^2^.

To gain a deeper understanding of the interaction between the defect and the skyrmion, system energy diagrams were calculated and are shown in Figure 4. The spin-texture snapshots along the energy lines are shown in Figure S1–S8 in the Supplementary Materials. The energy barriers and forces can be obtained from the system energy diagrams and other methods such as the GNEB method [40]. Firstly, we analyzed the case of a non-magnetic defect. For comparison, Figure 4a shows the system energy without a defect, in which the system energy first remains constant when the elliptical skyrmion is far away from the racetrack boundary, and then increases when the elliptical skyrmion is approaching the racetrack boundary, indicating that the racetrack boundary exerts a repulsive force on the skyrmion (the specific location of the skyrmion can be obtained from Figure S1). Figure 4b shows that, when the elliptical skyrmion approaches a non-magnetic defect (the skyrmion path and deformation are shown in Figure S2), the system energy increases, indicating that the non-magnetic defect exerts a repulsive force on the elliptical skyrmion outside the defect. Under the balance of the driving force from *J* and the repulsive forces from the defect and the racetrack boundary, the elliptical skyrmion is finally pinned at the top-left corner of the defect. 

Then, we analyzed the system energy under the influence of a magnetic defect. The role of *D_d_* of the magnetic defect was first studied, with *K_d_* remaining the same as *K_u_* (0.8 MJ/m^3^) of the racetrack and *J* remaining at a medium level of 5 MA/cm^2^. Figure 4c (corresponding to Figure S3) shows that, when *D_d_* is small and equals to 1.0 mJ/m^2^, the energy barrier in this case is 0.054 eV, indicating that the force exerted on the elliptical skyrmion outside the magnetic defect is repulsive. The energy barrier in this case is smaller than that (0.058 eV) in the case of the non-magnetic defect shown in Figure 4b, indicating that the repulsive force from the magnetic defect is smaller than that from the non-magnetic defect. Therefore, instead of being pinned at the top-left interior of the defect, the elliptical skyrmion can bypass the defect and then move away from the defect, resulting in Mode 1 in Figure 3a. Figure 4d shows that, when *D_d_* increases to 3.1 mJ/m^2^ (<*D_x_* = 3.7 mJ/m^2^), the energy barrier becomes smaller (0.026 eV), indicating that the repulsive force on the skyrmion outside the defect becomes smaller. The corresponding spin-texture snapshots are shown in Figure S4. This repulsive force is not large enough such that the elliptical skyrmion can approach and partly enters the defect. Interestingly, during the process of the elliptical skyrmion entering the defect, the system energy decreases, indicating that the defect exerts an attractive force on the elliptical skyrmion in the defect. Eventually, the elliptical skyrmion is pinned at the top-right interior of the magnetic defect owing to the balance of the driving force from *J* and the attractive force from the defect and the repulsive force from the racetrack boundary, resulting in Mode 2 in Figure 3b. Figure 4e shows that, when *D_d_* increases to 4.5 mJ/m^2^ (>*D_x_* = 3.7 mJ/m^2^) (Figure S5 shows the spin-texture snapshots in this case), the system energy decreases when the elliptical skyrmion approaches and enters the defect (1–1.7 ns), indicating that the defect shows an attractive force on the elliptical skyrmion no matter if it is outside or inside the defect, which then remains constant after the skyrmion gets pinned in the center of the defect (after 1.7 ns) due to the balance between the attractive force and driving force, resulting in Mode 3 in Figure 3c. In summary, in the case of *D_d_* < *D_y_*, the magnetic defect shows a repulsive (attractive) force on the elliptical skyrmion outside (inside) the defect, while in the case of *D_d_* > *D_x_*, the defect shows an attractive force on the elliptical skyrmion no matter if the elliptical skyrmion is outside or inside the defect.

The role of *K_d_* of a magnetic defect was then studied by using the system energy diagrams, with *J* remaining at 5 MA/cm^2^ and *D_d_* remaining at 3.1 mJ/m^2^. Figure 4f (corresponding to Figure S6) shows that, when *K_d_* < *K_u_*, the motion of the elliptical skyrmion is the same (Mode 3) as in the situation of *D_d_* > *D_x_*, indicating that the defect shows an attractive force on the elliptical skyrmion no matter if the skyrmion is outside or inside the defect. Figure 4g shows that, when *K_d_* is close to *K_u_* but still smaller than *K_u_*, the system energy first decreases as the elliptical skyrmion enters the defect, indicating that the defect shows an attractive force on the elliptical skyrmion and then oscillates as the elliptical skyrmion keeps trying to enter the defect, reflecting the complicated variation between the attractive force and the repulsive force, which is probably related to the difference between the elliptical shape of a skyrmion produced by anisotropic DMI constants and the circular shape of a defect with isotropic DMI constants. The specific location and shape of the skyrmion are shown in Figure S7. Since the force on the skyrmion is not always attractive and not strong enough to hold the skyrmion, the elliptical skyrmion can go through the defect center and escape, resulting in Mode 4 of the skyrmion. When *K_d_* > *K_u_*, as shown in Figure 4h and Figure S8, the system energy is similar to that when *D_d_* < *D_y_*, and the motion of the elliptical skyrmion is the same (Mode 1) as that when *D_d_* < *D_y_*, indicating that the defect shows a repulsive force on the elliptical skyrmion outside the defect. In short, in the case of *K_d_* > *K_u_*, the defect shows a repulsive force on the elliptical skyrmion outside the defect, while in the case of *K_d_* < *K_u_*, the defect shows an attractive force on the elliptical skyrmion no matter if the defect is outside or inside the defect.

**Figure 5:**
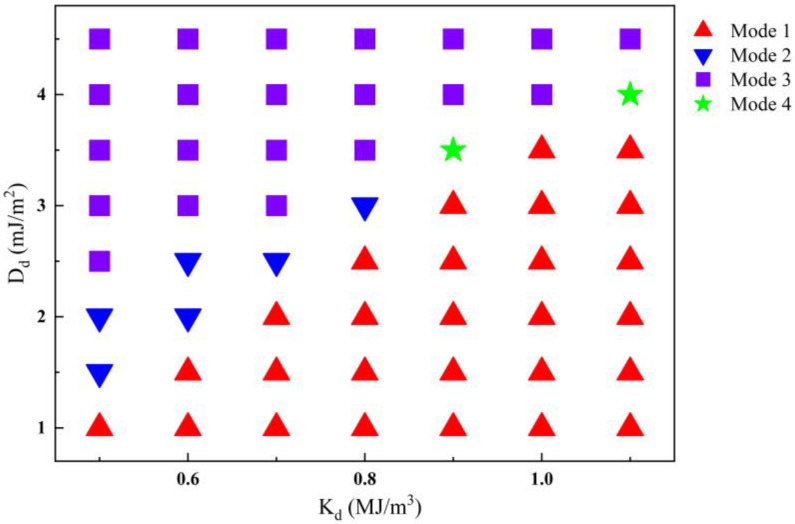
A phase diagram of elliptical skyrmion motion modes, showing the dependence of the elliptical skyrmion motion modes on *K_d_* and *D_d_*.

Then, the synergetic influence of *K_d_* and *D_d_* of the magnetic defect on the elliptical skyrmion was investigated by drawing motion-mode phase diagrams of the elliptical skyrmion, in which *J* remains at 5 MA/cm^2^ and the diameter *d* of the defect still remains to be 40 nm. As shown in Figure 5, the motion-mode phase diagram can be divided into three regions, i.e., *K_d_* < *K_u_*, *K_d_* = *K_u_* (0.8 MJ/m^3^), and *K_d_* > *K_u_*. In the case of *K_d_* = *K_u_*, when *D_d_* is smaller than 3.0 mJ/m^2^, the repulsive force on the elliptical skyrmion outside the defect is large enough and, hence, the skyrmion cannot touch the defect, resulting in Mode 1 of the elliptical skyrmion. When *D_d_* increases to 3.0 mJ/m^2^, the repulsive force gets smaller and cannot prevent the elliptical skyrmion from partly entering the defect, resulting in Mode 2 of the skyrmion. When *D_d_* increases further and is bigger than 3.0 mJ/m^2^, the force on the elliptical skyrmion outside the defect becomes attractive, and hence, the elliptical skyrmion enters the defect and is pinned at the center of the defect, resulting in Mode 3 of the skyrmion. In the region of *K_d_* < *K_u_*, when *D_d_* is a small value, the repulsive force on the elliptical skyrmion outside the defect is large enough, resulting in Mode 1 of the elliptical skyrmion. When *D_d_* increases to be close to *D_y_*, Mode 2 of the skyrmion appears since the repulsive force gets smaller and cannot prevent the elliptical skyrmion from touching the defect. When *D_d_* increases further and is bigger than *D_y_*, the motion of the elliptical skyrmion turns to Mode 3, indicating that the attractive force is dominant in this case. In the region of *K_d_* > *K_u_*, when *D_d_* is smaller than 3.0 mJ/m^2^, the motion of the elliptical skyrmion is obviously Mode 1 due to the large repulsive force on it in this case. As *D_d_* increases further, the elliptical skyrmion can go through the defect (Mode 4) because the force direction changes from being repulsive to attractive from the perspective of *D_d_*. Finally, when *D_d_* = 4.5 mJ/m^2^, the attractive force on the elliptical skyrmion in the defect becomes dominant such that the skyrmion gets pinned at the center of the defect (Mode 3).

**Figure 6:**
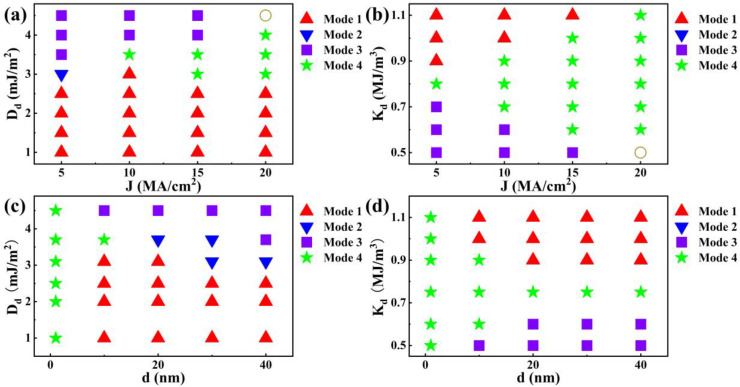
Phase diagrams of elliptical skyrmion motion modes, showing the dependence of the elliptical skyrmion motion modes on (**a**) the driving current density *J* and *D_d_*; (**b**) *J* and *K_d_*; (**c**) the diameter of the defect *d* and *D_d_*; and (**d**) *d* and *K_d_*. An open circle means that the elliptical skyrmion is annihilated in the defect.

In the following paragraphs, the influences of the driving current density *J* and the magnetic defect size *d* on the dynamics of the elliptical skyrmion are investigated. Firstly, the synthetic influence of *J* and *D_d_* on the motion of the elliptical skyrmion is investigated and shown in Figure 6a. The diameter of the magnetic defect remains at 40 nm. In the region of *D_d_* < 3.0 mJ/m^2^, the motion of the elliptical skyrmion is always Mode 1 when the driving current *J* increases from 5 MA/cm^2^ to 20 MA/cm^2^, indicating that the defect exerts a large repulsive force such that the elliptical skyrmion cannot touch the defect. In the case of *D_d_* = 3.0 mJ/m^2^, when *J* equals to 5 MA/cm^2^, the motion of the elliptical skyrmion is Mode 2 because the repulsive force from the defect decreases with increasing *D_d_* such that part of the skyrmion can go inside the defect and then get pinned at the top-right interior of the defect; when *J* increases to *J* = 10 MA/cm^2^, the motion of the elliptical skyrmion changes to Mode 1 because the driving force from *J* increases such that the skyrmion can be propelled away from the pinning position; when *J* increases further to *J* = 15 MA/cm^2^ and 20 MA/cm^2^, the motion of the elliptical skyrmion changes to Mode 4 because the driving force from *J* is so much bigger than the repulsive force that the skyrmion feels when it is outside the defect and the attractive force that the skyrmion feels when it is inside the defect. As a result, the skyrmion is first forced into the defect, then is forced out of the defect, and finally escapes. Eventually, in the region of *D_d_* > 3.0 mJ/m^2^, the motion of the elliptical skyrmion changes from Mode 3 to Mode 4 as *J* increases from 5 MA/cm^2^ to 20 MA/cm^2^, depending on the competition of the driving force from *J* and the repulsive (attractive) force the skyrmion feels when it is outside (inside) the defect, which varies with the *D_d_* value. It is noted that, when *D_d_* = 4.5 mJ/m^2^ and *J* = 20 MA/cm^2^, the elliptical skyrmion will be annihilated due to the larger driving force from *J* and the larger attractive force from the defect with respect to those in the situations when *D_d_* = 4.5 mJ/m^2^ and *J* = 15 MA/cm^2^ and when *D_d_* =4.0 mJ/m^2^ and *J* = 20 MA/cm^2^.

Figure 6b shows the synthetic influence of *J* and *K_d_* on the motion of the elliptical skyrmion. In the region of *K_d_* < 0.8 MJ/m^3^, when *J* increases from 5 MA/cm^2^ to 20 MA/cm^2^, the motion of the elliptical skyrmion changes from Mode 3 to Mode 4, depending on the competition of the driving force from *J* and the attractive force that the elliptical skyrmion feels, which varies with the *K_d_* value. Similarly, when *K_d_* = 0.5 mJ/m^2^ and *J* = 20 MA/cm^2^, the elliptical skyrmion will be annihilated due to the tearing between the force from the driving current and the defect. Then, in the region of *K_d_* > 0.8 MJ/m^3^, when *J* increases from 5 MA/cm^2^ to 20 MA/cm^2^, the motion of the elliptical skyrmion changes from Mode 1 to Mode 4, indicating that the elliptical skyrmion can eventually overcome the repulsive force from the defect with increasing *J*. 

Then, we investigated the size effect of the magnetic defect. The current density remains to be 5 MA/cm^2^. Figure 6c shows the synthetic influence of the defect diameter *d* and *D_d_* on the motion of the elliptical skyrmion. Interestingly, when *d* = 1 nm, the motion of the elliptical skyrmion is always Mode 4 no matter what the value of *D_d_* is because the defect is so tiny that the skyrmion can easily go across it. When *d* varies between 10 nm and 40 nm, the motion of the elliptical skyrmion is dependent on the value of *D_d._* When *D_d_* is small and not larger than 2.5 mJ/m^2^, the motion of the elliptical skyrmion is always Mode 1 because the repulsive force from the defect is large enough such that the skyrmion cannot touch the defect no matter what its diameter is. When *D_d_* becomes bigger and equals to 3.1 mJ/m^2^, the repulsive force from the defect gets smaller and the motion of the skyrmion is dependent on the defect size: the skyrmion goes across when the defect size is small and not larger than 20 nm, but it gets stuck in the top-right interior when the defect size is bigger than 20 nm. When *D_d_* increases to 3.7 mJ/m^2^, the result is similar to that of the situation when *D_d_* = 3.1 mJ/m^2^, but the minimum size of the defect for the appearance of Mode 2 decreases to 20 nm, and Mode 3 appears when *d* = 40 nm due to the larger attractive force. When *D_d_* is big and equals to 4.5 mJ/m^2^, the attractive force from the defect becomes so large that the elliptical skyrmion gets stuck at the center of the defect no matter what the defect size is. Similarly, Figure 6d shows the synthetic influence of *d* and *K_d_* on the motion of the elliptical skyrmion. When *d* = 1 nm, the motion of the elliptical skyrmion always belongs to Mode 4 no matter what the value of *K_d_* is due to the tiny size of the defect. As *d* varies between 10 nm and 40 nm, the motion of the elliptical skyrmion is dependent on the value of *K_d_* as follows: In the case of *K_d_* = 0.5 MJ/m^3^, the motion of the elliptical skyrmion is always Mode 3 for the reason that the attractive force from the defect is so large that the elliptical skyrmion gets pinned at the center of defect no matter what the defect size is. As *K_d_* increases to 0.6 MJ/m^3^, the minimum size of the defect for the appearance of Mode 3 increases to 20 nm due to the smaller attractive force. As *K_d_* = 0.75 MJ/m^3^, the attractive force from the defect becomes even smaller and the motion of the elliptical skyrmion is always Mode 4 owing to the minor difference between the defect and the racetrack. Eventually, when *K_d_* is big and equals to 1.1 MJ/m^3^, the motion of the elliptical skyrmion always belongs to Mode 1 since the repulsive force from the defect is large enough such that the skyrmion cannot touch the defect no matter what its diameter is. 

**Figure 7:**
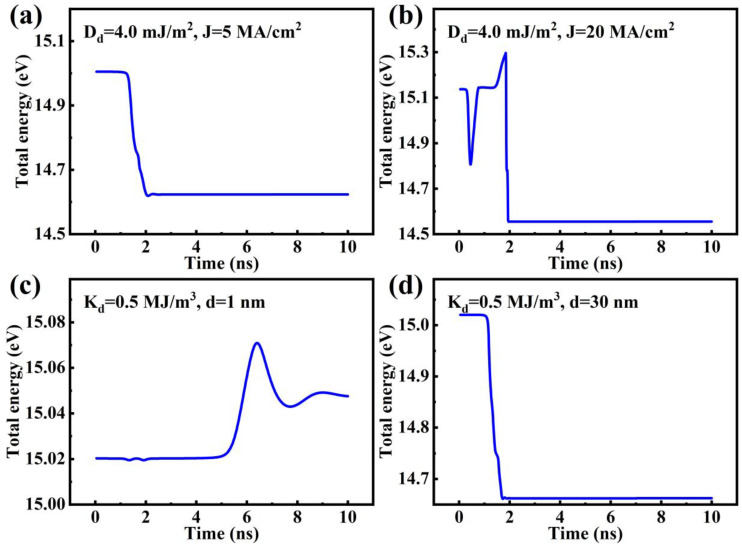
The system energy of every step time when (**a**) *D_d_* = 4.0 mJ/m^2^ and *J* = 5 MA/cm^2^; (**b**) *D_d_* = 4.0 mJ/m^2^ and *J* = 20 MA/cm^2^; (**c**) *K_d_* = 0.5 MJ/m^3^ and *d* = 1 nm; (**d**) *K_d_* = 0.5 MJ/m^3^ and *d* = 30 nm.

To gain a deeper understanding of the pinning and depinning discussed above, Figure 7 shows the energy diagrams of two points in Figure 6a to display the influence of *J* and of two points in Figure 6d to display the influence of *d* (corresponding spin-texture snapshots are shown in Figures S9–S11). In the case of *D_d_* = 4.0 mJ/m^2^, when *J* = 5 MA/cm^2^, the system energy decreases as the elliptical skyrmion approaches and enters the defect (Figure 7a), indicating that the defect shows an attractive force on the elliptical skyrmion, but when *J* increases to 20 MA/cm^2^, the elliptical skyrmion can overcome the energy barrier (0.338 eV) and escape from the defect owing to the larger driving force (Figure 7b). The different energy barriers in Figure 7a,b originated from the changing shapes of the skyrmion. In the case of *K_d_* = 0.5 MJ/m^3^, when *d* = 1 nm, the energy barrier is so small that the elliptical skyrmion can go across the defect directly (Figure 7c). But when the size of the defect increases to 30 nm, the energy barrier gets larger and the skyrmion gets pinned (Figure 7d). By comparing Figure 7c,d, one can see that a larger size of the defect will cause a larger energy valley and result in a larger attractive force on the elliptical skyrmion. 

**Figure 8:**
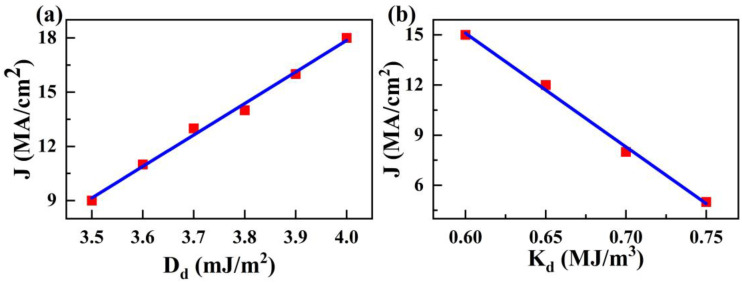
(**a**) The minimum driving current density *J*, as a function of *D_d_*. (**b**) The minimum driving current density *J*, as a function of *K_d_*.

Finally, since the performance of the racetrack memory device is significantly influenced by the pinned skyrmion, the depinning conditions for the completely pinned skyrmion in Mode 3 were investigated. The minimum driving current density *J* to free the pinned elliptical skyrmion was simulated for a 40 nm diameter magnetic defect with various values of *D_d_* and *K_d_*, as shown in Figure 8. The results indicate that the depinning minimum *J* for the elliptical skyrmion stuck in the defect linearly increases with increasing *D_d_* and decreases with increasing *K_d_* because the attractive force from the defect on the elliptical skyrmion increases with increasing *D_d_* and decreases with decreasing *K_d_*, respectively, as discussed above.

## 4. Conclusions

In this study, we systematically investigated the dynamics of an elliptical skyrmion driven by different current densities in a racetrack device in the presence of a circular-shaped defect with different magnetic parameters and sizes. It is found that the magnetic parameters play critical roles in determining the type (repulsive or attractive) and the magnitude of the force on the elliptical skyrmion. When the DMI constant of the defect is larger than that of the racetrack or the PMA constant of the defect is smaller than that of the racetrack, the defect exerts an attractive force on the elliptical skyrmion. And when the DMI constant of the defect is smaller than that of the racetrack or the PMA constant of the defect is larger than that of the racetrack, the defect exerts a repulsive force on the elliptical skyrmion. The magnitude of the force depends on the difference between the magnetic parameters of the defect and the racetrack. Dependent on the values of *D_d_*, *K_d_*, and *d* of the defect and the driving current density *J*, the primary motion modes of the elliptical skyrmion in the defective racetrack can be divided into four types. By analyzing the motion-mode phases, the synthetic effects of *D_d_*, *K_d_*, *d*, and *J* are illustrated. Finally, the minimum depinning current density *J* is investigated for a completely pinned skyrmion, which is useful for restarting the normal operation of a racetrack memory device with unavoidable defects. Our findings bridge the gap between the motion modes of an elliptical skyrmion and the interactions in a defective racetrack, which can be used to improve the performance of a defective racetrack memory device by utilizing the dynamics of an elliptical skyrmion.

## Data Availability

The data presented in this study are available on request from the corresponding author.

## Supplementary Materials

The following supporting information can be downloaded at: https://www.mdpi.com/article/10.3390/nano14030312/s1, Figures S1–S8: The spin-texture snapshots along the energy lines corresponding to Figure 4; Figures S9–S12: The spin-texture snapshots along the energy lines corresponding to Figure 7.
