# Supplementary material for: Dynamics of Elliptical Magnetic Skyrmion in Defective Racetrack
Source: Nanomaterials (Basel). 2024 Feb 4;14(3):312. doi: 10.3390/nano14030312 (PMC10857043; doi:10.3390/nano14030312)
Supplement: Supplementary file 1 [file nanomaterials-14-00312-s001.zip › nanomaterials-2824808-supplementary.pdf]

Supplementary Materials

Dynamics of Elliptical Magnetic Skyrmion in Defective Racetrack

Huangkun Zhu, Gang Xiang, Youhua Feng, Xi Zhang†,

[1]*College Of Physics, Sichuan University, Chengdu 610065, China*

†E-mail address: xizhang@scu.edu.cn

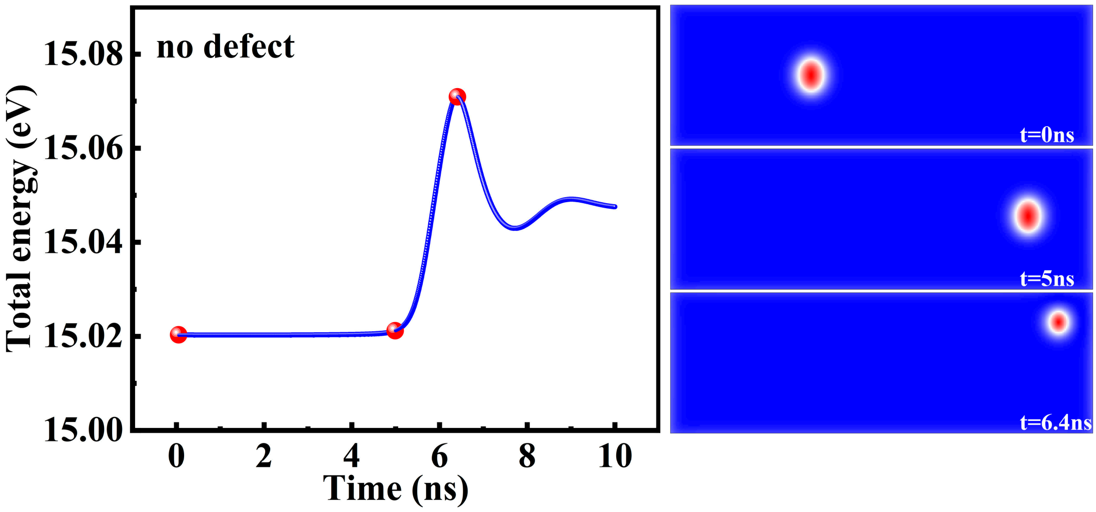

Figure S1 The spin-texture snapshots along the energy lines in the case of no defect, which correspond to the red points in the energy diagram, respectively.

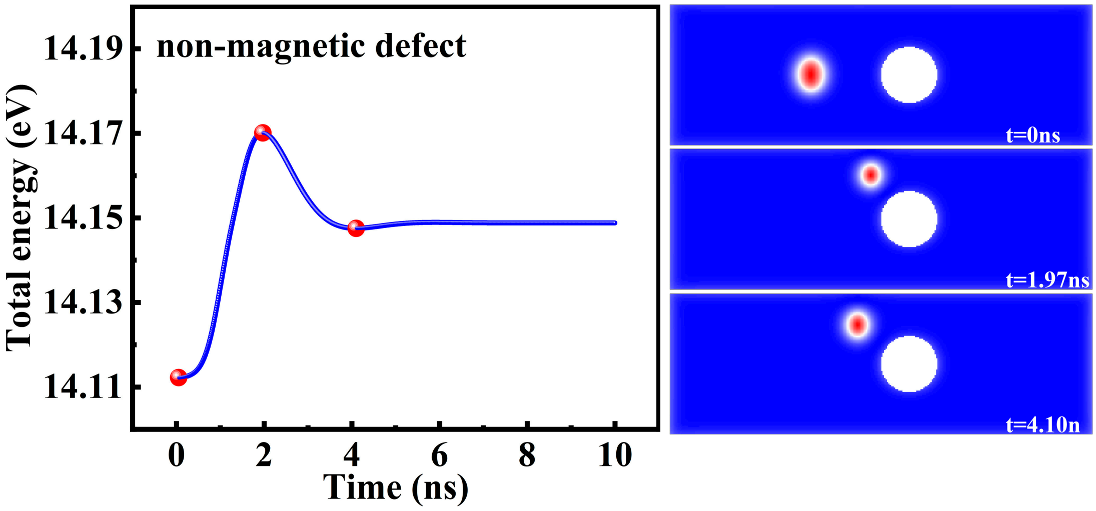

Figure S2 The spin-texture snapshots along the energy lines in the case of non-magnetic defect, which correspond to the red points in the energy diagram, respectively.

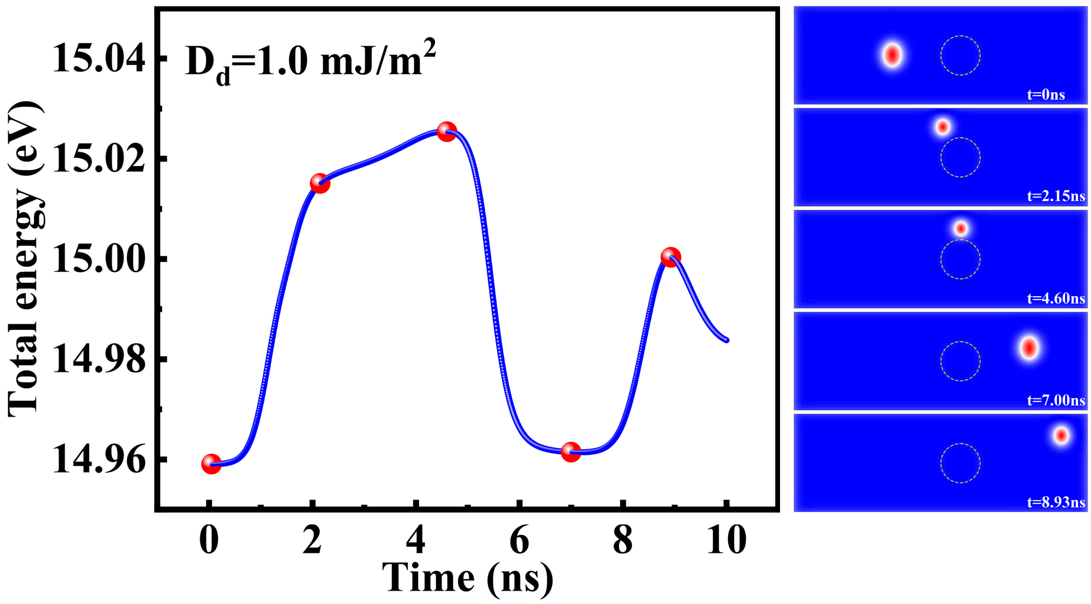

Figure S3 The spin-texture snapshots along the energy lines when D_d=1.0 mJ/m^2, which correspond to the red points in the energy diagram, respectively. The yellow circle represents the defect position.

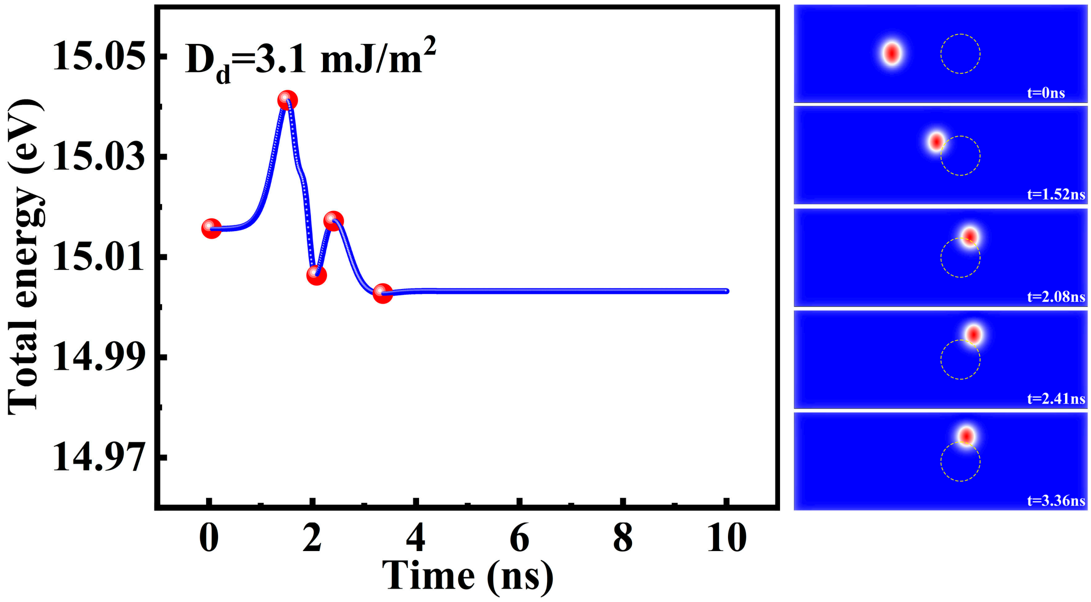

Figure S4 The spin-texture snapshots along the energy lines when D_d=3.1 mJ/m^2, which correspond to the red points in the energy diagram, respectively. The yellow circle represents the defect position.

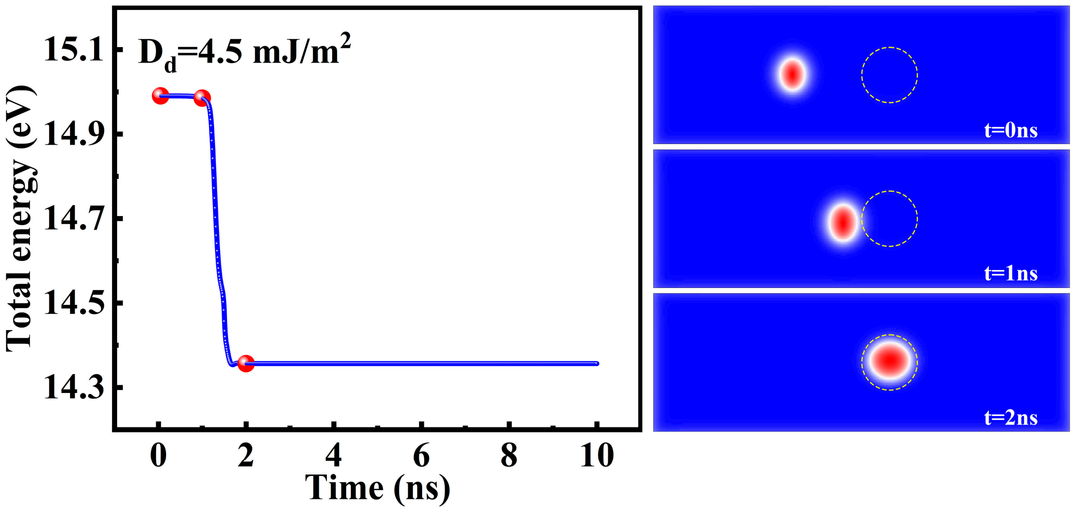

Figure S5 The spin-texture snapshots along the energy lines when D_d=4.5 mJ/m^2, which correspond to the red points in the energy diagram, respectively. The yellow circle represents the defect position.

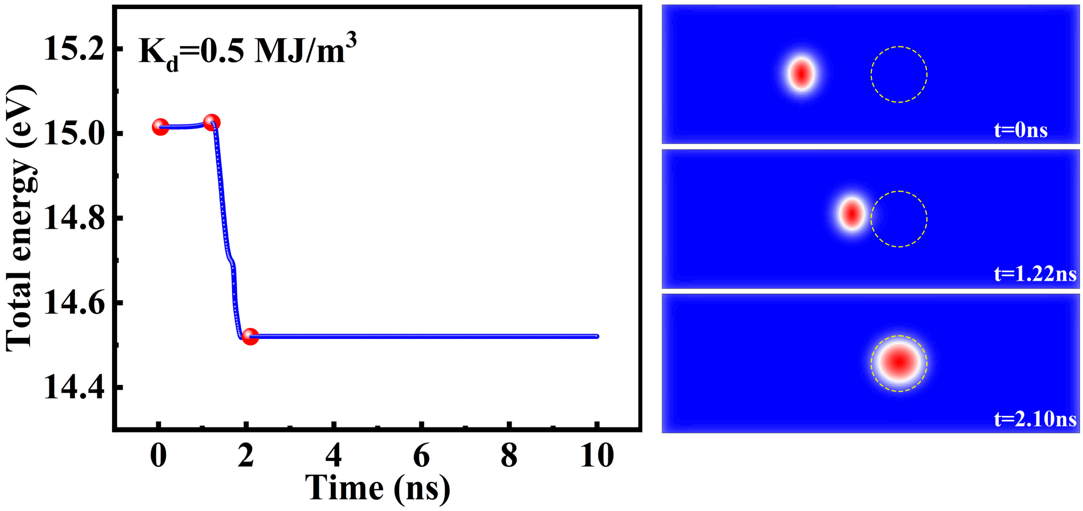

Figure S6 The spin-texture snapshots along the energy lines when K_d=0.5 MJ/m^3, which correspond to the red points in the energy diagram, respectively. The yellow circle represents the defect position.

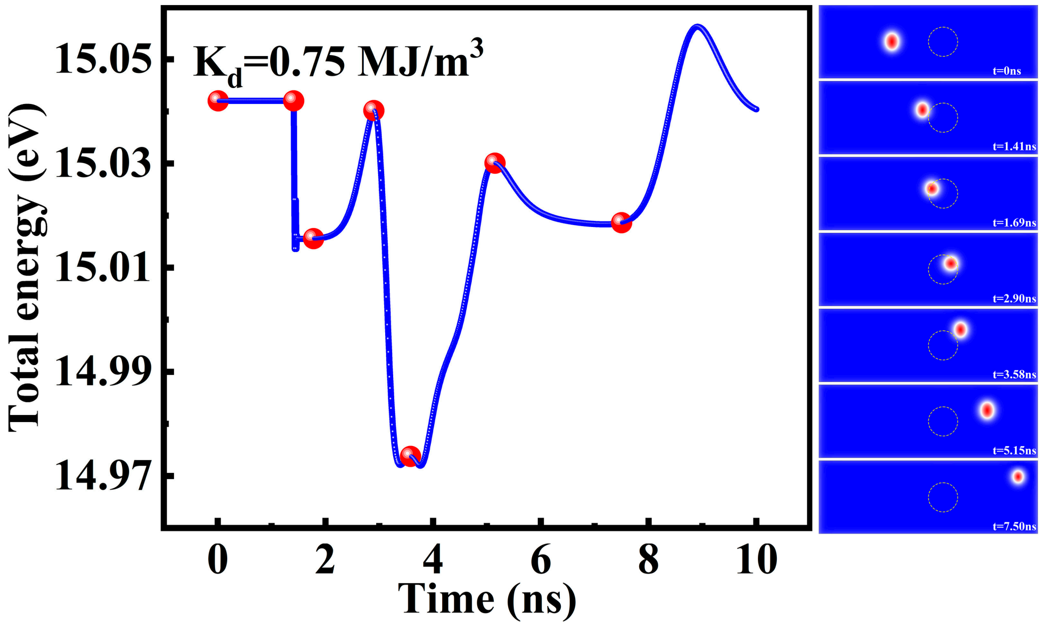

Figure S7 The spin-texture snapshots along the energy lines when K_d=0.75 MJ/m^3, which correspond to the red points in the energy diagram, respectively. The yellow circle represents the defect position.

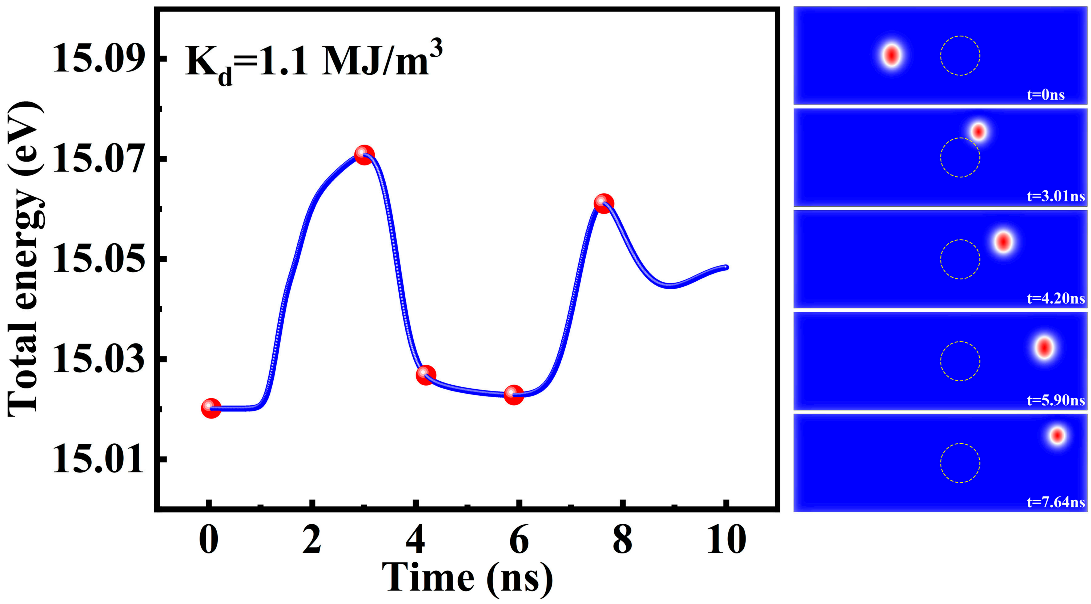

Figure S8 The spin-texture snapshots along the energy lines when K_d=1.1 MJ/m^3, which correspond to the red points in the energy diagram, respectively. The yellow circle represents the defect position.

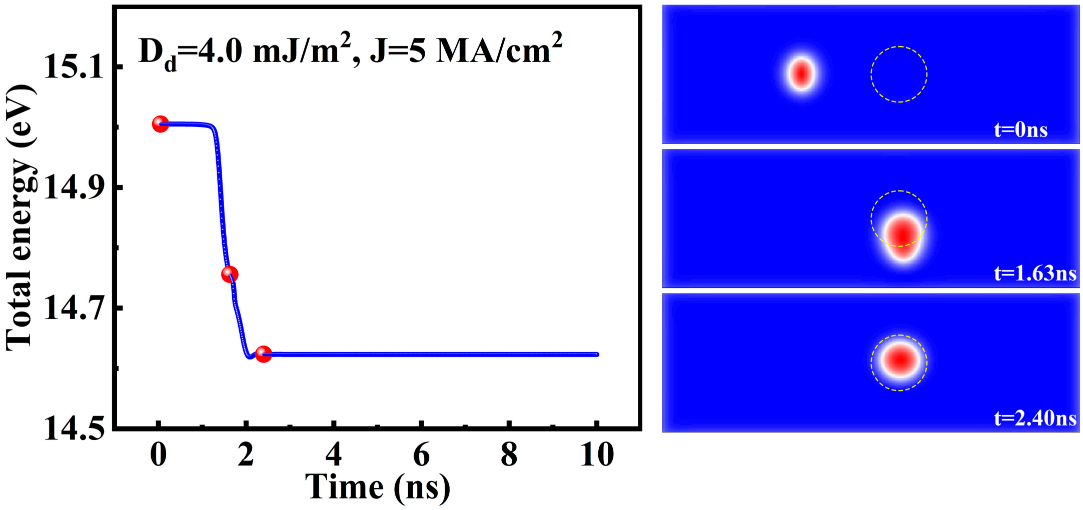

Figure S9 The spin-texture snapshots along the energy lines when D_d=4.0 mJ/m^2 and J=5 MA/cm^2, which correspond to the red points in the energy diagram, respectively. The yellow circle represents the defect position.

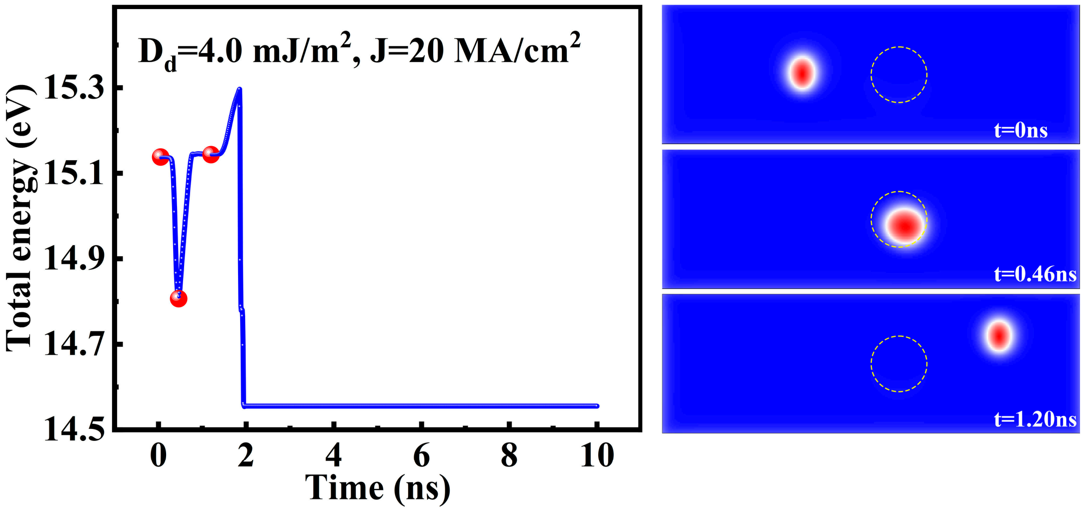

Figure S10 The spin-texture snapshots along the energy lines when D_d=4.0 mJ/m^2 and J=20 MA/cm^2, which correspond to the red points in the energy diagram, respectively. The yellow circle represents the defect position.

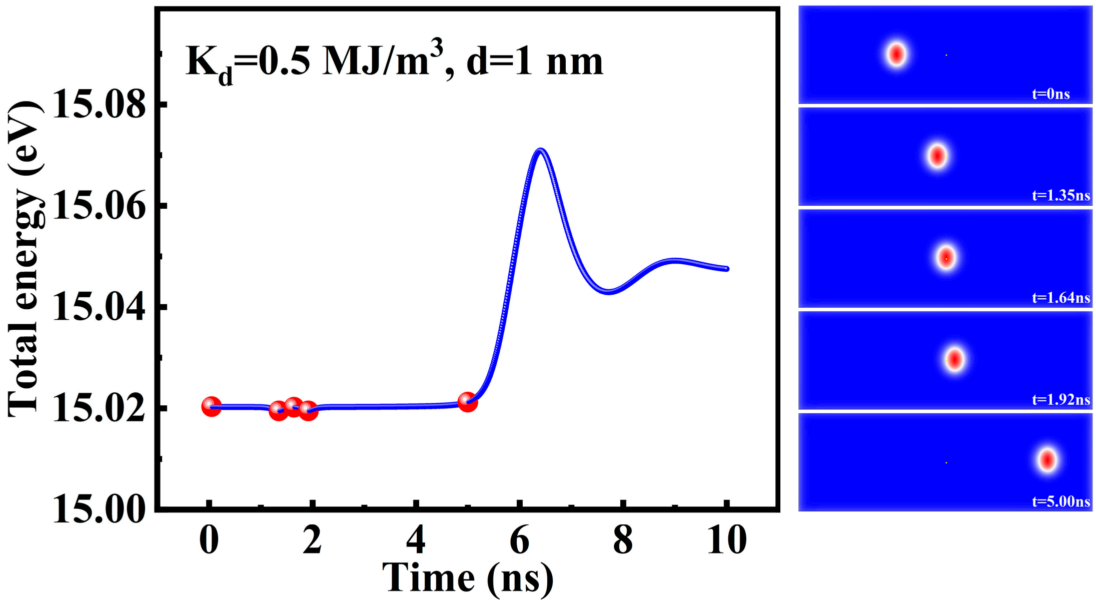

Figure S11 The spin-texture snapshots along the energy lines when K_d=0.5 MJ/m^3 and *d*=1 nm, which correspond to the red points in the energy diagram, respectively. The yellow circle represents the defect position.

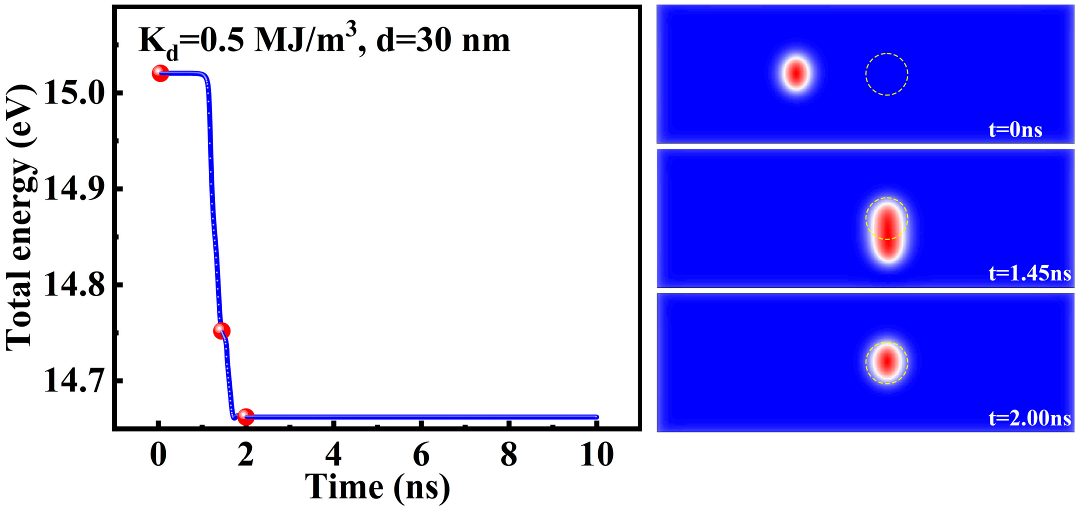

Figure S12 The spin-texture snapshots along the energy lines when K_d=0.5 MJ/m^3 and *d*=30 nm, which correspond to the red points in the energy diagram, respectively. The yellow circle represents the defect position.
